# Supplementary figures and images for: Murine cytomegaloviruses m139 targets DDX3 to curtail interferon production and promote viral replication
Source: PLoS Pathog. 2020 Oct 8;16(10):e1008546. doi: 10.1371/journal.ppat.1008546 (PMC7575108; doi:10.1371/journal.ppat.1008546)

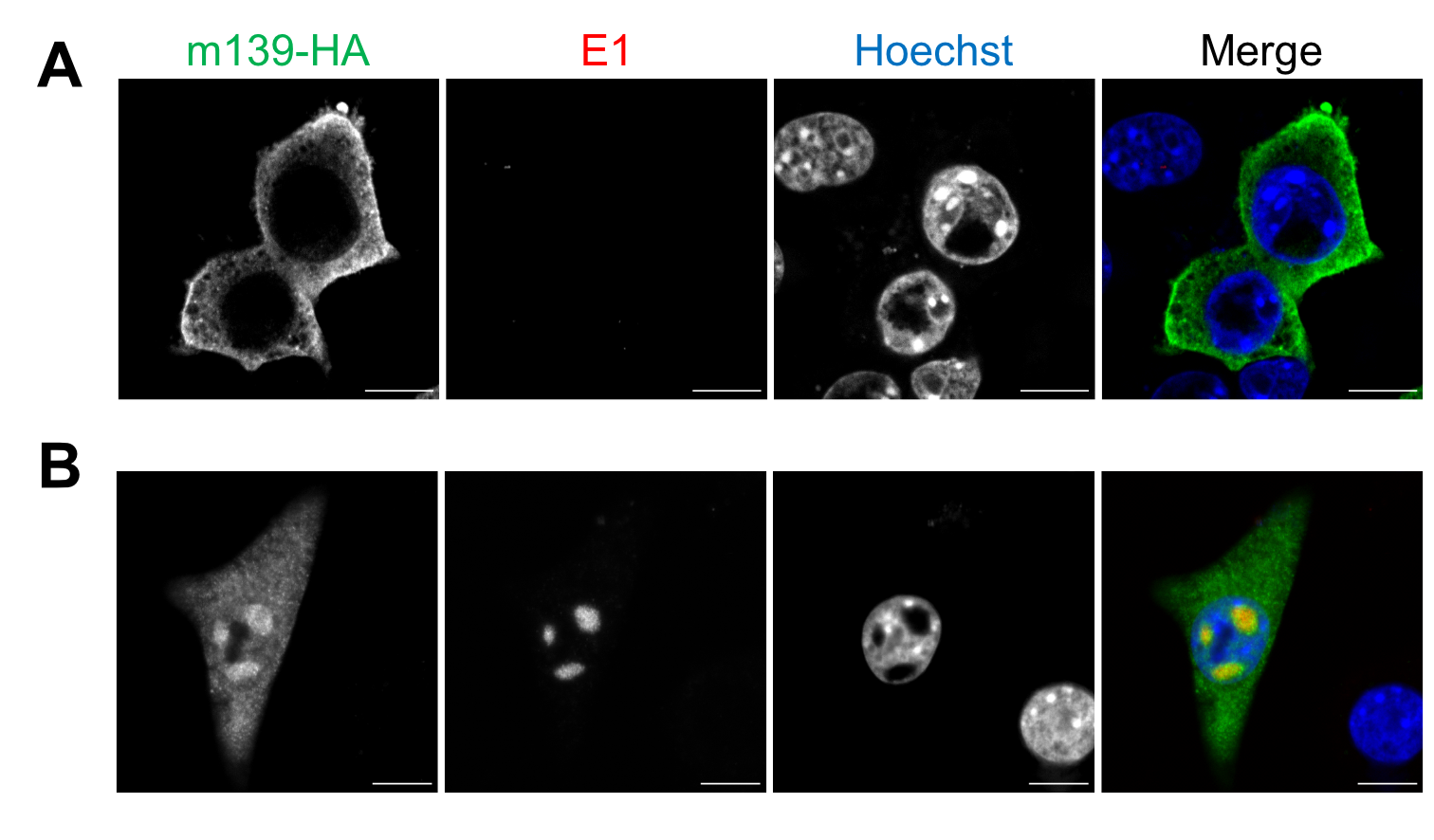

Supplement: S1 Fig — NIH-3T3 fibroblasts were transfected with a plasmid encoding HA-tagged MCMV m139 (A) or plasmids encoding m139 and the MCMV E1 proteins (B). Cells were fixed 24 hpi and analyzed by immunofluorescence using antibodies specific for the HA epitope tag and E1. Nuclei were stained using Hoechst 33342. Images were obtained by confocal laser scanning microscopy and are representative of three independent experiments. Scale bar, 10 μm. (TIFF) [file ppat.1008546.s001.tiff]

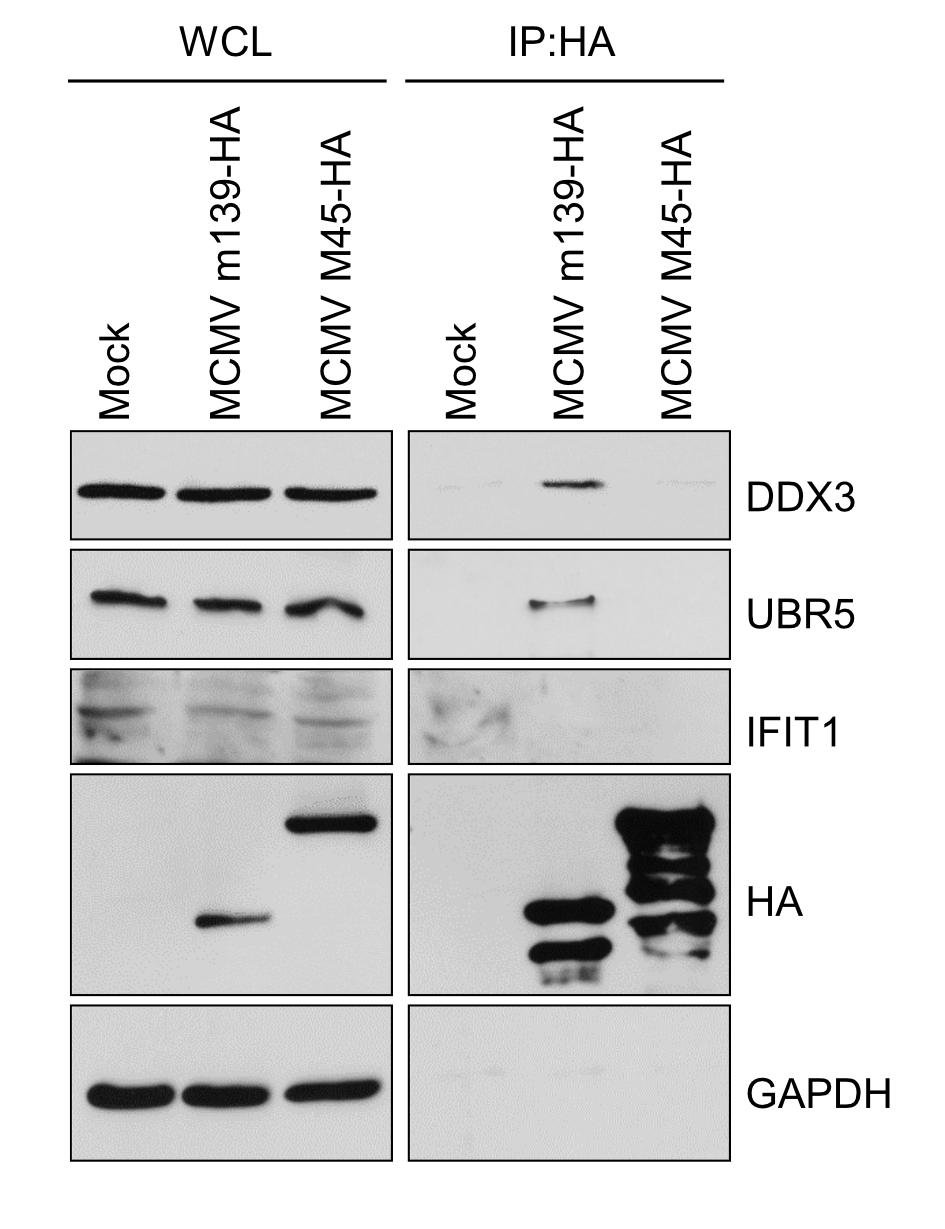

Supplement: S2 Fig — iBMDM were infected with MCMV m139-HA or M45-HA at an MOI of 5. Cell lysates were collected 24 hpi and subjected to immunoprecipitation (IP) using an anti-HA affinity matrix. Co-precipitating proteins were detected by immunoblotting with specific antibodies. (TIFF) [file ppat.1008546.s002.tiff]

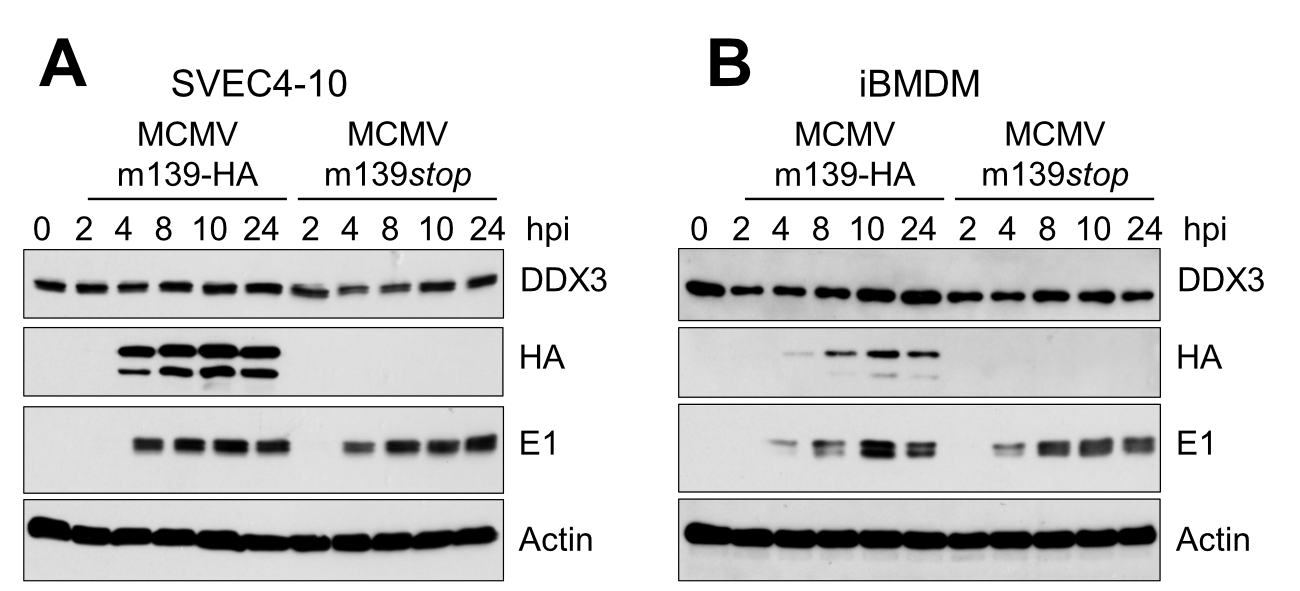

Supplement: S3 Fig — (A) SVEC4-10 cells and (B) iBMDM were infected with MCMV m139-HA or MCMV m139stop at an MOI of 5. Whole cell lysates were prepared at the indicated times post infection and analyzed by immunoblot analysis. (TIFF) [file ppat.1008546.s003.tiff]

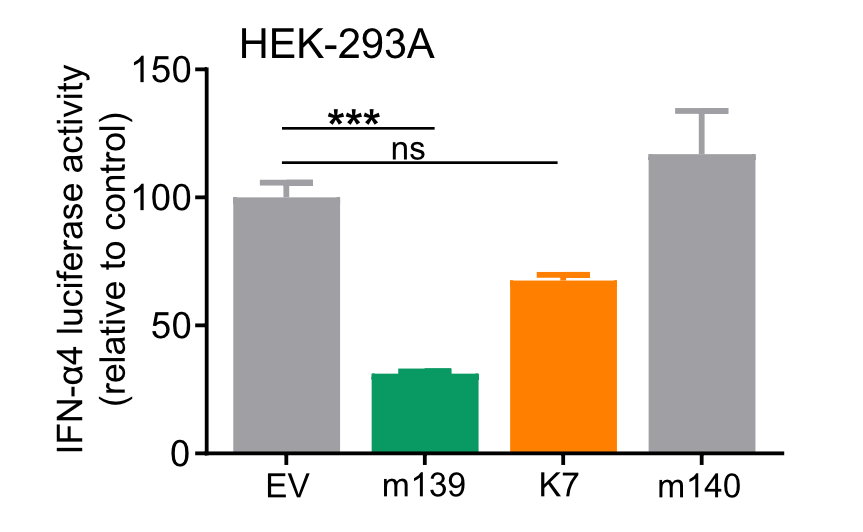

Supplement: S4 Fig — HEK-293A cells were co-transfected with DDX3 and IRF7(2D) expression plasmids, an IFNα4-luc reporter plasmid, a renilla luciferase normalization control. Plasmids expressing MCMV m139, m140, VACV K7, or empty vector (EV) were co-transfected. IRF7(2D) is a constitutively active IRF7. Firefly and renilla luciferase activities were determined in the same samples. Values were normalized to those of cells co-transfected with EV. Means ±SD of three biological replicates are shown. The result is representative of three independent experiments. (TIFF) [file ppat.1008546.s004.tiff]

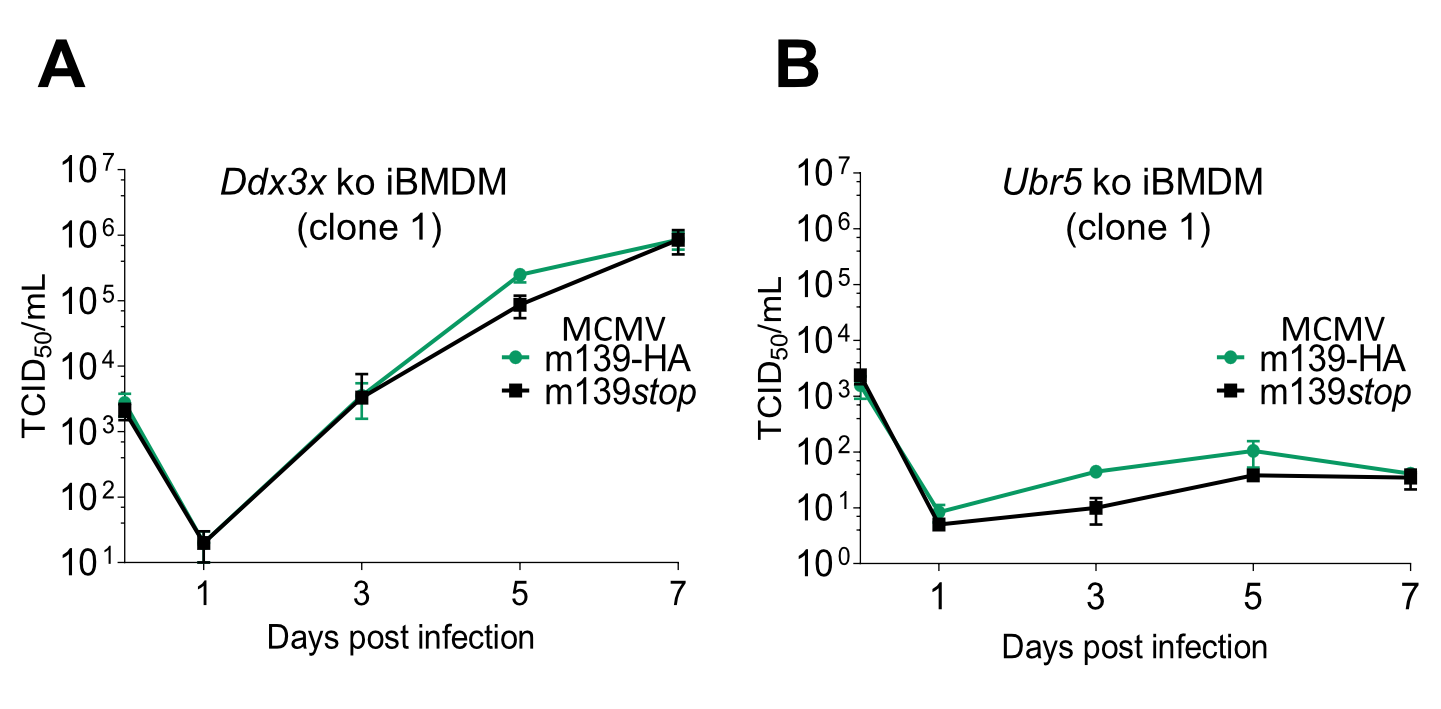

Supplement: S5 Fig — (A) Ddx3x ko iBMDM (clone 1) or (B) Ubr5 ko iBMDM (clone 1) were infected with MCMV m139-HA or m139stop (MOI = 0.025). Virus release into the supernatant was quantified by titration. Viral titers are shown as means ±SD of three biological replicates. (TIFF) [file ppat.1008546.s005.tiff]
