# Supplementary material for: Murine cytomegaloviruses m139 targets DDX3 to curtail interferon production and promote viral replication
Source: PLoS Pathog. 2020 Oct 8;16(10):e1008546. doi: 10.1371/journal.ppat.1008546 (PMC7575108; doi:10.1371/journal.ppat.1008546)
Supplement: Attachment — Submitted filename: Rebuttal.pdf [file ppat.1008546.s006.pdf]

Point-by-point response

Editor comment:

The three reviewers were consistent in their enthusiasm for your manuscript. However, there were also three common concerns that are evident in their reviews that would certainly need addressing in a revised manuscript. These were: 1) the need to further solidify the link between m139 and IFN expression, 2) required consolidation of the in vivo data (also with investigation of the relationship between m139 and IFN in vivo) and 3) the utility of UBR5 and/or DDX3 deficient cells in m139-stop rescue experiments to further solidify the relationship between these viral and host proteins.

Response: We thank the editors for their guidance and for specifying which experiments are essential and need to be included in a revised manuscript. As described in detail in the point-by-point response to the reviewers' comments, we have

(1) solidified the link between m139 and IFN expression. As suggested by reviewer #1, we have measured IFN-α and IFN-β production as well as additional cytokines (TNFα, IL-6, and CXCL10) by macrophages infected with MCMV-m139HA or m139stop. The results were included in Figure 6. We also confirmed the interaction of m139 with DDX3 and UBR5 in MCMV-infected macrophages (new Fig S2) as suggested by reviewer #2.

(2) consolidated the in vivo data by analyzing more time points (3, 7, and 14 dpi) and organs (popliteal lymph nodes, spleen, liver, lungs, salivary glands). The data are shown an expanded Figure 8 and described in the associated text (lines 400-407). We have also measured INF-α and IFN-β levels in serum and spleen of infected mice and found increased IFN-α and IFN-β levels in mice infected with the m139stop mutant. The new data are included in Figure 6.

(3) further solidified the relationship between m139 and DDX3 and UBR5 by knocking out Ddx3x or Ubr5 in iBMDM. We show that the Ddx3x knockout rescued the replication defect of the m139stop mutant in iBMDM, while MCMV replication in Ubr5 knockout iBMDM was generally impaired. As suggested by reviewer #1, we also investigated viral replication in type I IFN receptor (Ifnar) knockout macrophages and found no replication defect of the m139stop mutant, suggesting that the replication defect in WT iBMDM is IFN-dependent. The results are included in Figure 7.

Apart from these three essential revisions, we have also addressed the remaining reviewer comments as explained below in the point-by-point response.

Reviewer #1:

The manuscript by Puhach and co-workers extends studies on the murine cytomegalovirus (MCMV) protein m139, a member of the US22 gene family previously shown to be required for efficient viral replication in macrophages, and to be a potential inhibitor of IFN-beta induction. Here, the authors provide mechanistic insights into how m139 achieves that. They report that this viral protein interacts with the host DEAD box helicase DDX3 and with the E3 ubiquitin ligase UBR5 to promote viral replication in macrophages and endothelial cells. They demonstrate that via DDX3, the m139 protein inhibits IFN-

beta production in macrophages, while in endothelial cells the increased replication associated to DDX3 and UBR5 is not due to a blockade of IFN-beta induction. Finally, they describe that, in vivo, m139 is key to viral dissemination to local lymph nodes and to the salivary glands. The manuscript is properly organized, well executed, and reports interesting results. While virally-encoded DDX3 inhibitors that dampen interferon production have been already described, m139 represents the first example found in herpesviruses. Thus, the study describes an important additional strategy of host innate immune evasion used by MCMV.
However, there are some relevant issues that are not addressed in the manuscript at present. Does m139 inhibit IFN-beta production in vivo? The interaction of m139 with DDX3/UBR5 probably leads to the alteration of additional cellular targets besides IFN-beta? This is an important aspect that needs to be determined.

Response: We thank this reviewer for appreciating our study and for suggesting experiments to further strengthen the manuscript.

Major issues

1. Since DDX3 and UBR5 are enzymes involved in numerous host processes, participating in the regulation at different levels of cellular mRNAs, most likely the effects of the interaction of m139 with these two host components are not limited to the inhibition of IFN-beta. Taking this in consideration, an analysis of the m139 effects on selected DDX3/UBR5 targets should be performed. Is IFN-alpha production also altered by m139? Anti-inflammatory cytokines? Related to this issue: the authors nicely show that the replication defect of MCMV m139 stop is rescued in DDX3- and in UBR5-deficient cells. Following this same line, can the replication defect of the mutant virus be reverted in IFNalphaRko iBMDMs? If this is the case, in which extent?

Response: Following this reviewers' suggestion we measured the production of IFN-α and IFN-β as well as additional cytokines (TNFα, IL-6, CXCL10) and ISG20 by macrophages infected with MCMV-m139HA or m139stop. We found that IFN-α and IFN-β production as well as the transcription of interferon-stimulated genes Cxcl10 and Isg20 was strongly upregulated in the absence of m139, whereas IL-6 and TNF-α were only slightly affected. The results were included in Figures 6 and 7. We also analyzed MCMV replication in newly generated Ddx3x and Ubr5 ko iBMDM as well as in Ifnar ko iBMDM. We found that replication of MCMV m139stop was rescued to wildtype levels in Ddx3x ko as well as in Ifnar ko iBMDM, suggesting that the replication defect was DDX3 and type I IFN dependent. The new results were included in Figure 7.

2. The authors demonstrate that the m139-deficient MCMV is compromised in its ability to replicate in vivo in popliteal lymph nodes and salivary glands. However, they haven't formally proven that the decreased in vivo growth of the mutant is due, at least in part, to an increase in IFN-beta levels. At a minimum, the levels of IFN-alpha and IFN-beta in serum and selected organs of mice infected with the m139-deficient MCMV should be analyzed and compared to those in MCMV infected mice under the same conditions. In addition, can the decreased replication of MCMV m139 stop observed in popliteal lymph nodes and salivary glands be extended to other key organs?

Response: To address this issue, we have measured IFN-α and IFN-β in sera and spleens of MCMV-infected mice. We found significantly increased IFN levels in mice infected with m139stop (see Figure 6). We also

determined viral titers in the spleen (day 3 and 7), liver, lungs (day 7), and SG (day 7 and 14) of infected mice. The PLN, lung, and SG titers are shown in Figure 8. The titers in spleen and liver were below the detection threshold for all three viruses (stated in the manuscript text, line 400-403), which is not unexpected considering the infection route (footpad) and dose.

3. The authors haven't aimed to identify the specific DDX3-dependent cellular process/es interfered by m139. The manuscript would benefit from the inclusion of assays addressing this issue, for example to discern whether the viral protein targets the interaction of DDX3 with IKK-epsilon or blocks DDX3 binding to the IFN-beta promoter.

Response: This reviewer raises an interesting question, which is not easily answered. In an attempt to address this question, we tested whether m139 inhibits activation of the IFN-α promoter as it has been shown for VACV K7. Using a previously described luciferase reporter assay we showed that m139 inhibits IFN-α promoter activation in HEK 293A cells co-transfected with DDX3 and IRF7 expression plasmids (Figure 6I). Interestingly, IFN-α promoter activation was also inhibited by m139 if cells were transfected with a plasmid expressing constitutively active IRF7 (see Fig S4), suggesting that m139 blocks IFN-α promoter activation downstream of IRF7 activation, possibly by inhibiting DDX3 binding to the promoter (line 329-336).

Minor issues

1. Fig. 6A should be quoted in the text. Eliminate "Error! Reference source not found" in l. 229.

Response: This mistake was corrected in the revised manuscript. Fig 6A is now referenced in the text.

2. The number of independent experiments performed need to be incorporated in some Figure legends.

Response: We have included the number of biological replicates and independent experiments in the figure legends as requested.

3. Black and white images in some of the panels in Figures 1C and 4 could be provided in color for a better visualization.

Response: We have used color for the merged images. For the individual stainings we prefer to keep the images B/W (greyscale) because the contrast is better. Particularly, red on black background has a very poor contrast when printed on paper.

4. Table 1, a legend to describe L/H H/L should be included.

Response: A legend describing the meaning of L (light) and H (heavy) amino acid labeling has been included.

Reviewer #2:

The manuscript by Puhach et al. deals with a novel molecule involved in the innate immunity against herpesviruses, and the respective antagonism by cytomegaloviruses. It uncovers important parallels to poxviruses and their Bcl2-like innate immune evasins.
The topic is highly relevant. The paper is nicely written and clear.

Response: We thank this reviewer for appreciating the relevance of our work and for suggestions on how to improve the manuscript.

Major issues

The manuscript follows two aspects:
(I) In an endothelial cell line (SVEC4-10), an interaction of the MCMV-encoded m139 with UBR5 and DDX3X as well as an UBR5- and DDX3X-dependent growth impairment of the m139-deficient MCMV mutant was uncovered.
(II) In the macrophage-like cell line NR-9456, m139 was found to be a functional analogue of (and to be functionally replaceable by) the VACV-encoded K7 in terms of IFNβ induction.
Given that K7 targets DDX3X in order to prevent IFNβ induction, both lines of evidence appear to converge nicely. However, the m139 coding capacity seems not affect the IFNβ induction in SVEC cells (although there seems be a trend in this direction) and the K7 insertion does not revert the replication of Δm139-MCMV in SVEC cells. Thus, the functional association of the m139-DDX3X interaction and the effect on IFNβ induction - as stated in the title - is so far not sufficiently substantiated by experimental evidence. Key experiments are:
(I) To test if m139-HA precipitates UBR5, DDX3X, and IFIT1 in NR-9456 cells.

Response: Following this reviewer's suggestion, we have repeated the immunoprecipitation experiment (Fig. 3A, done in SVEC4-10 cells) in iBMDM (=NR-9456 cells). The result is essentially the same as the one obtained in SVEC4-10 cells: DDX3 and UBR5, but not IFIT1, co-precipitated with m139 (shown in the new supplementary Fig S2).

(II) To test if an UBR5 and/or DDX3X knock-out /-down reverts the Δm139-MCMV replication in NR-9456 cells.

Response: Following this reviewer's suggestion, we generated Ddx3x and Ubr5 knockout iBMDM (NR-9456 cells) by CRISPR/Cas9 gene editing. As shown in Figure 7, MCMV-m139HA and m139stop replicated to the same titers in Ddx3x knockout iBMDMs. Both viruses also replicated to the same titers in Ubr5 ko cells, but viral replication was generally impaired, suggesting that the loss of Ubr5 is detrimental for MCMV replication in iBMDM. Thus, the Ddx3x knockout rescued the growth defect of the m139stop mutant, similar to the rescue previously shown in SVEC4-10 cells (Fig. 5).

Given that K7 binds a hell of proteins (PMID: 28815417), I would also check how Δm139-MCMV:K7 replicates in the SVEC clones either lacking UBR5 or DDX3X just to rule out that K7 causes some weird problems in this respect. SVEC cells are SV40-transformed cells. Couldn't the authors assess Δm139-MCMV induction and replication in primary mouse ECs (e.g., PMID: 28060318) or Luka's EC model (PMID: 23773211)?

Response: We agree that the DDX3 and UBR5-dependent attenuation of MCMV m139stop in SVEC4-10 cells deserves further investigation. Our results indicate that the attenuation in SVEC4-10 cells is likely not IFN-mediated. Almost all requests from the three reviewers focused on m139 and its effect on IFN production in macrophages and in mice, which is also the main focus of the manuscript. Therefore, we

focused our efforts on this most important aspect of the story. A mechanistic investigation of the m139-dependent phenotype in SVEC and other endothelial cells will have to await future studies.

Honestly, the in vivo analysis is a bit premature. Some more organs and time points should be assessed.

Response: A similar request was made by reviewer #1. For the revised manuscript, we also determined viral titers in the spleen (day 3 and 7), liver, lungs (day 7), and salivary glands (day 7 and 14) of infected mice. The PLN, lung, and SG titers are shown in Figure 8. The titers in spleen and liver were below the detection limit for all three viruses (stated in the manuscript text, line 400-403), which is not unexpected considering the infection route (footpad) and dose.

Minor issues

To line 227: IFNβ induction by MCMV was previously shown for a macrophage cell line by Le et al. (PMID: 18420790) and for primary macrophages by Döring et al. and Ehlting et al. (PMIDs: 25231302 & 26299622). These papers should be cited.

Response: Thank you. We were happy to include these references.

Interactions of CMV-encoded proteins with UBR5 have been shown before (PMID: 30619335, PMID: 21320693). These papers should be discussed.

Response: Thank you. We discussed these papers in the revised manuscript (line 490).

My quick alignment revealed that m139 harbours two short stretches (DTTYTLVREYITFR and DTAGELTPLGVCA) with certain sequence homology to the VACV-encoded proteins K7 and A52. Intriguingly, these similarities are either overlapping or close to regions, which are involved in DDX3X binding described in the following article (PMID: 19913487). More specific mutations might help to sort things out.

Response: Thank you for taking the time to do this alignment. We mention a possible structural similarity of m139 and K7 in the discussion (line 440).

SEM is not a descriptive statistics and should not be used as such. The replication curves and luciferase data sets should be depicted as mean (or median) ± standard deviation (SD).

Response: In the revised version, we changed the relevant figures and used SD instead of SEM.

In line 229, there is a problem with the reference in the database.

Response: This mistake has been corrected.

In Fig.2A, there seems to be an additional symbol (a black triangle?) at the 7 dpi point.

Response: We have checked this figure and the underlying data set to make sure that there are no additional symbol.

All error bars in the replication curved should be depicted in a colour different from the curve and the data points in order to allow the reader to appreciate it.

Response: We have changed the error bars from SEM to SD as requested by this reviewer. The error bars are now better visible.

Reviewer #3:

Puhach et al. addressed the function of the murine cytomegalovirus open reading frame m139. To this end the authors generated a recombinant virus expressing HA-tagged m139. They detected early kinetics of m139 expression. Obviously the viral E1 protein recruits m139 to pre-replication compartments in the nucleus. Studies with cell lines revealed that m139 is required for efficient MCMV replication in macrophages and endothelial cells, whereas it is dispensable in fibroblasts and epithelial cells. m139 interacts with the viral m140 and m141 proteins, but not E1, and with the host components DDX3, UBR5 and IFIT1. Replication of m139stop virus was rescued in DDX3 as well as UBR5-deficient cells suggesting that DDX3 and UBR5 are restriction factors of MCMV replication, which are counteracted by m139. Furthermore, the interaction of m139 with DDX3 seems to depend on UBR5 and vice versa. Finally, m139 inhibits DDX3-dependent IFN-beta induction and m139 is required efficiently infect distal sites such as popliteal lymph nodes and salivary glands.

The authors have chosen a straight forward approach to study the function of m139, generation of recombinant viruses either expressing HA-tagged m139 or m139stop. This is a solid study that is based on overall well controlled experiments. The data are interesting for virologists specialized on MCMV. The presented data further illuminate the complex interactions of viral components with host factors.

Response: We thank this reviewer for his/her insightful comments, which helped us to improve the manuscript.

Major issues

Usually, VSV is a very potent inhibitor of IFN responses. Thus, the statement in line 232/233 could be misleading. Please indicate which VSV strain was used.

Response: We used VSV-GFP, a recombinant VSV strain that was used previously for the induction of IFN-β transcription (PMID 14585354). This information was included in the revised manuscript (legend to Fig 6), and a reference was included in the Materials and Methods section.

The control experiment that replication of MCMV m139stop can be rescued in DDX3 ko or UBR5 ko iBMDM should be included.

Response: Following this reviewer's suggestion, we generated Ddx3x and Ubr5 knockout iBMDM by CRISPR/Cas9 gene editing. As shown in Figure 7D, MCMV-m139HA and m139stop replicated to the same titers in Ddx3 knockout iBMDMs. Thus, the knockout rescued the growth defect of the m139stop mutant, similar to the rescue previously shown in SVEC4-10 cells (Fig. 5).

The conclusions in line 13/14 and line 283/284 are formulated too strong. Since M139 is required for efficient replication at local sites it is well possible that the reduced local virus titers are not sufficiently high in order to support virus transport to secondary target organs, especially the salivary glands. This would results in diminished MCMV titers in the salivary glands as shown in Fig. 8. Thus, if no other experiments are presented it remains unclear whether reduced local replication of MCMV m139stop or reduced dissemination is the underlying mechanism. Importantly, in this study it was not addressed whether m139 deficiency somehow affects the transporting myeloid cells itself.

<u>Response</u>: We agree that it is difficult to distinguish between reduced virus transport to and reduced virus replication within target organs. Both can result in reduced titers in target organs and thus affect dissemination. However, we understand from this comment that the term "dissemination" leaves room for interpretation and could be interpreted to mean transport. Therefore, we have added a paragraph to the discussion to explain that dissemination to distant sites depends on both, local replication and transport to distant sites by myelomonocytic cells. We also point out that the experiments shown in this paper do not allow us to discriminate between reduced transport and reduced local replication.

Minor issues

In line 229 a reference is missing.

In line 24 the word "cell" is missing.

In the title of Fig. 1 the word "the" is duplicated.

<u>Response</u>: These three errors have been corrected. Thank you.
